# Autogenous transplantation of mandibular third molar to replace tooth with vertical root fracture

**Published:** 2009-07-06

**Authors:** Saeed Asgary

**Affiliations:** 1 *Department of Endodontics, Iranian Center for Endodontic Research, Dental Research Center, Dental School, Shahid Beheshti University of Medical Sciences, Tehran, Iran*.

**Keywords:** Autogenous tooth transplantation, Autotransplantation, CEM cement, New material, NEC, Third molar

## Abstract

Autogenous tooth transplantation (ATT) can be considered when there is a hopeless molar tooth and suitable donor present. This report presents an unconventional case of successful ATT of a third molar replacing the adjacent fractured second molar in a 33 year old woman. This wisdom tooth had completely developed roots. Root-end filling with Calcium Enriched Mixture (CEM) cement was performed in the third molar. The second molar was extracted non-traumatically without any bone removal; the wisdom tooth was immediately transplanted into the recipient socket. No endodontic treatment was carried out either during or after the ATT. At six-month and 2-year clinical examination the patient was asymptomatic; the transplanted tooth was still functional, with no evidence of marginal periodontal pathosis. At the same follow ups, radiographic evaluation illustrated bone regeneration, normal PDL, and absence of external root resorption. Transplantation of mature third molar seems to be a promising method for replacing a lost permanent molar tooth and restoring aesthetics and function.

## INTRODUCTION

Dental autotransplantation or autogenous tooth transplantation (ATT) was first reported in 1951 (1). ATT is defined as the transplantation of teeth from one site to another in the same person (2). The recipient site may be either an extracted-tooth socket or a surgically-prepared alveolus. ATT is a viable treatment for the replacements of a traumatized tooth when there is an available donor tooth (3).

Loss of molar teeth may have different etiologies *i.e.* untreatable large decay, severe periodontal disease, failed root canal treatment and root fractures/perforations (4). From a clinical point of view, ATT of a third molar for replacement of an untreatable first or second molar tooth is an occasionally appropriate alternative to the usual prosthetic rehabilitation or implant treatments (5). With little or no external root resorption, a transplanted third molar is able to maintain natural space, alveolar bone volume, and the morphology of the alveolar ridge through proprioceptive stimulation (6). Appropriate treatment planning accompanying good surgical method causes high rates of success for ATT (7). It has been reported that the success rate may be excellent if the donor third molar has been transplanted before complete root formation (8), however, successful ATTs of teeth with complete root development has been well recognized (9). Probability of higher success rate of ATT has been also reported for close-apex teeth (10-12). Therefore, ATT is feasible for molar teeth with closed apices, but endodontic treatment is usually indicated for such teeth (11).

The outcome of ATT depends on wise case selection and considerion of all biological aspects. The critical event is the preservation of PDL cellular vitality in aseptic conditions. Healing of the PDL is critical for survival of the transplanted tooth; ankylosis may accur if the tooth is not transplanted in an hour. Achievement of a good apical seal is an essential factor for having normal function (13).

**Figure 1:**
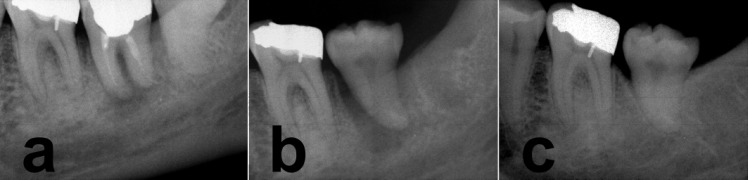
a) Initial periapical radiograph. The second mandibular tooth had vertical root fracture and chronic apical periodontitis. The third mandibular tooth was healthy, b) Radiographic image immediately after transplantation and c) Two years after transplantation, bone and PDL regeneration was observed. Root resorption was not observed.

Recently, a novel endodontic material (NEC) named calcium enriched mixture (CEM) cement has been developed as root-end filling material (14). Although *in vitro* studies on sealing ability of CEM, and MTA, as the gold standard (14-15), and the *in vivo* vital pulp therapies on animals and humans (16-18) revealed comparable results, CEM cement offers better physical properties than MTA (19). This dental material is able to form hydroxyapatite over its surface in normal saline (20) and does exhibit similar characteristics to the surrounding dentine when used as root-end filling (21). Additionally, it has a lower estimated cost.

The aim of this case report is to demonstrate a successful replacement of an untreatable second mandibular molar with vertical root fractured with its adjacent completely-developed third mandibular molar.

## CASE REPORTS

A 33-year-old Caucasian woman was referred to an endodontic practice for extraction of her mandibular left second molar. The medical history was non-contributory. Clinical examination revealed good oral hygiene but the second molar had vertical fracture. Periodontal probing depths did not exceed 3 mm, however, the involved tooth was tender to percussion and palpation. The radiographic examination showed previous endodontic treatment with poor instrumentation and unsatisfactory obturation. A large extended periapical lesion was observed. The patient was informed about the fractured tooth; and extraction was recommended to her.

The examinations also showed that adjacent third molar was healthy, favourably developed, completely erupted and well positioned within the dental arch, and therefore it was an appropriate candidate for ATT (Figure 1a). The treatment procedures in addition to benefits and risks of the technique were explained to the patient. A written informed consent was obtained and the patient was scheduled for treatment.

At the treatment session, oral disinfection was carried out with 0.2% chlorhexidine gluconate solution. Surgery was performed under local anaesthesia with a mandibular block (2% lidocaine and 1:80000 epinephrine; Daroupakhsh, Tehran, Iran). First, the fractured second molar and then the wisdom tooth were extracted. The third molar was then positioned into the recipient socket to assess its adaptation. As a result of the small size of single donor root, adequate adaptation was achieved with ease.

Root-end resection was made by removing 3mm of the tooth root apex. Three millimetres deep class I root-end preparations were made using an ultrasonic power unit (miniPiezon, EMS, Nyon, Switzerland) with ultrasonic retrotips (DT-043, EMS, Nyon, Switzerland) and irrigated with sterile normal saline solution. The root-end cavity were dried with absorbent paper points and filled with CEM cement. The tooth was rinsed in sterile saline to remove all debris and was then replaced in recipient socket with favourable distance (1 mm) to the adjacent teeth and no interference with opposing teeth (Figure 1b). The transplanted tooth was fixed with silk sutures. It only took 12 minutes to extract the tooth and then to transplant it (extraoral time).

**Figure 2:**
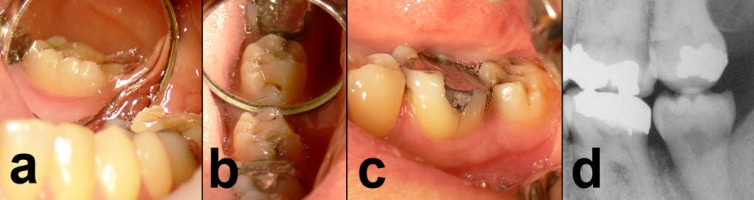
a, b and c) Lingual, occlusal and buccal aspects of transplanted tooth after six months; clinically, the tooth showed no sensitivity to percussion and presented normal occlusion, periodontal conditions and masticatory function and d) Bite-wing radiograph six months after transplantation illustrated good occlusal interrelationship with opposite tooth.

The patient was recalled for clinical and radiographic follow-ups after 1 day (post-operative check-up), 1 week (suture removal), 1 month (clinical examination), 6 and 24 months (clinical and radiographic examinations). The postoperative period was uneventful. Figures 2a-d and 1c show the radiographic and clinical features of the transplanted third molar at 6 and 24 month follow-ups.

At the 2-year follow up visit the tooth had normal occlusion, with physiological mobility and masticatory function. Periodontal probing revealed no pockets or other pathological signs, and the patient was symptom-free. The periodontal ligament appeared intact; periradicular area had normal status and no evidence of root resorption or periapical lesion was observed.

## DISCUSSION

Teeth with vertical root fractured and poor prognosis are classified as a genuine indication for ATT (6). In the present case, the patient presented an unsatisfactory endodontic treatment, chronic pain, and sensitivity to percussion and palpation in second left mandibular molar. Such tooth sockets can be considered a suitable candidate as a recipient site for ATT. On the other hand, an intact mature third mandibular molar adjacent to the involved tooth with no function due to missing of opposite upper third molar provided us an acceptable donor graft. However, only a few reports have been published regarding the outcome of ATT of third molars with closed apices, the majority reported favourable outcomes (10,11,22,23).

It is obvious that ATT requires size compatibility between the transplanted tooth and recipient socket. In the present case, the third molar was single-rooted which was smaller than recipient site. Thus, extraoral modification of the donor root was not required and the transplanted tooth was simply inserted, well positioned and easily stabilized in the new site. It was reported that close contact of the transplanted tooth with the alveolar bone of the recipient site may provide better blood supply for the PDL cells (24). Although the transplanted tooth in the present study was poorly adapted to the recipient site and exhibited great mobility, wound healing and absence of clinical signs/symptoms of ankylosis, pain or sensitivity to percussion were confirmed at all the follow-ups. Also, radiographs illustrated the presence of periodontal ligament along the entire root surfaces and absence of periapical lesion associated with the tooth root.

The critical factor for inflammatory root resorption after ATT is the infection of root-canal system. ATT of closed-apices teeth, therefore, necessitates pulp extirpation within 1 to 2 weeks to avoid pulpal infection followed by periradicular inflammation and subsequent inflammatory root resorption (25). This seems to be justified by the fact that only 15% of teeth with closed apices were revitalized after ATT, in contrast with 96% of teeth with open apices (26). In this reported case, root canal therapy was not carrying out during or after transplantation. Root-end filling was used instead of RCT as the transplanted tooth had only one main root canal, which can easily be treated apically. Utilization of CEM cement as root-end filling material is expected to favour bone repair and inhibit inflammatory root resorption due to its favourable sealing ability (14,15), high pH (19), antibacterial effect (27-29) and biocompatibility (16,17,30).

The favourable results obtained in this rare case of ATT may be attributed to distinct factors, such as maintaining asepsis during surgical procedure, atraumatic surgical extraction and replacement, preservation of the PDL cellular vitality, minimal extraoral time, good occlusal interrelationship, adequate fixation as well as filling and sealing the apex with a biocompatible root-end filling material.

ATT of third molars with closed apices may be considered as a viable treatment option compared to the usual prosthetic and implant treatments for both restorative and financial reasons. 
